# Editorial on Special Issue “Medical Data Processing and Analysis”

**DOI:** 10.3390/diagnostics13122081

**Published:** 2023-06-16

**Authors:** Wan Azani Mustafa, Hiam Alquran

**Affiliations:** 1 Faculty of Electrical Engineering Technology, Campus Pauh Putra, Universiti Malaysia Perlis, Arau 02600, Malaysia; 2 Advanced Computing (AdvComp), Centre of Excellence (CoE), Universiti Malaysia Perlis, Arau 02600, Malaysia; 3 Department of Biomedical Systems and Informatics Engineering, Yarmouk University, Irbid 21163, Jordan; heyam.q@yu.edu.jo

Medical data plays an essential role in several applications in the medical field. Medical data can be raw data, signals, images, and several observations. The processing and analysis of the data lead to inferring many vital points that help in diagnosis, treatment, progression, and decision-making. Various research handles the medical data in many aspects, such as improving time forecasting models using machine learning for future pandemic applications based on COVID-19 data 2020–2022. The validated proposed method outperformed existing models in both accuracy and efficiency. The authors highlighted the significance of machine learning techniques in predicting the spread of pandemics, in which the proposed model can be exploited for future pandemic applications. The study emphasizes the importance of accurate forecasting models in managing pandemics and highlights the limitations of traditional statistical models in capturing the complex dynamics of pandemics. The designed model overcomes limitations and is considered a standalone public health system [1]. However, it requires the employment of deep learning to extract deep features from white blood cells to classify them into different categories [2]. The optimized entropy-controlled deep features are utilized to build a prominent model that performs success over all existing models in various terms of sensitivity, specificity, and accuracy [2]. The medical data can be transformed using algorithms such as features transformation to predict blood glucose in type 1 diabetes mellitus patients. H. Butt et al. [3] proposed technique outperforms existing accuracy, sensitivity, and specificity methods. They also discussed the potential implications of the proposed technique for improving the management of T1DM and developing more accurate and efficient medical diagnostic tools. Valuable insights were also provided into the current state of research in the field and identified potential avenues for future research [3]. Moreover, using deep learning with medical images improves the detection of *H. pylori* atrophic gastritis. Apart from that, Yasmeen Yacoob et al. [4] proposed a deep learning-based approach for detecting atrophic gastritis caused by Helicobacter pylori (*H. pylori*) infection. The proposed method utilizes an enhanced convolutional neural network (CNN) learner to extract features from endoscopic images of the gastric mucosa and classify them into normal or abnormal categories. The proposed method aims to improve the accuracy of existing detection methods and reduce the need for invasive diagnostic procedures such as biopsy. They explore the impact of accurate detection of *H. pylori*-related atrophic gastritis for preventing and treating gastric cancer, a common complication of chronic *H. pylori* infection. The proposed method is evaluated using a dataset of endoscopic images from patients with *H. pylori* infection. It is compared with other state-of-the-art methods for atrophic gastritis detection. The results show that the proposed method outperforms existing methods in accuracy, sensitivity, and specificity [4].

ECG signals can be represented as images using various transformation techniques. A. Zyout et al. employed a combination of wavelet and Hilbert–Huang transforms to extract relevant features from ECG signals and apply a support vector machine (SVM) classifier to diagnose them. They evaluate the proposed method on a publicly available MIT–BIH arrhythmia database and compare the results with existing ECG wave recognition methods. The proposed method achieved an overall accuracy of 99.25% in recognizing ECG waves, significantly higher than the existing methods. The authors further analyze the performance of the proposed method in identifying individual ECG waves, such as P-waves, QRS complexes, and T-waves. The results show that the proposed method achieves high accuracy in recognizing all the ECG waves, with an average accuracy of 99.4% for P-waves, 99.9% for QRS complexes, and 99.6% for T-waves. The high accuracy achieved in recognizing individual ECG waves is crucial for diagnosing and treating specific cardiac conditions [5]. On the other hand, A. Almulihi et al. [6] proposed an ensemble learning approach based on a hybrid deep learning model for early heart disease prediction. The authors combine a CNN and a long short-term memory (LSTM) network to capture both spatial and temporal features of electrocardiogram (ECG) signals. The proposed method is evaluated on the publicly available PTB diagnostic ECG database and compared with existing ECG-based heart disease prediction models. The results show that the proposed hybrid deep learning model outperforms existing models concerning the accuracy, sensitivity, specificity, and area under the receiver operating characteristic curve (AUC-ROC). The proposed model achieves an accuracy of 95.96%, a sensitivity of 95.85%, a specificity of 96.07%, and an AUC-ROC of 0.9909, which are significantly higher than the existing models. Their proposed method has the potential for clinical use in the early diagnosis and treatment of heart disease [6].

The protein sequence data are exploited to detect COVID-19 and influenza viruses. M. Erten et al. used a hamlet-pattern-based method to extract features from the protein sequences and then apply an SVM classifier for detection. The proposed model is evaluated on several datasets, including the SARS-CoV-2, influenza virus, and non-viral protein datasets. The proposed model achieves an accuracy of 99.61% and an AUC-ROC of 1.00 in detecting the COVID-19 virus and an accuracy of 99.66% and an AUC-ROC of 1.00 in detecting the influenza virus. The model also shows high specificity in detecting viral proteins, with a specificity of 99.99% for COVID-19 and 99.96% for influenza viruses. The proposed model can be used in clinical settings for early detection and diagnosis of COVID-19 and influenza viruses [7]. However, the genomic and tabular data with recurrent neural networks are employed to predict type-2 diabetes. P. N. Srinivasu et al. [8] proposed the RNN model utilizing DNA sequence (as genetic data) and tabular data, such as patient characteristics and medical history. The evaluation is performed on a dataset of patients with and without type-2 diabetes, and the results show that it outperforms other state-of-the-art models in terms of accuracy, sensitivity, specificity, and AUC-ROC. The model achieves an accuracy of 88.1%, a sensitivity of 84.8%, and a specificity of 91.4% in predicting the risk of type-2 diabetes. The AUC-ROC score of the proposed model is 0.935, indicating a high discriminative power in distinguishing between patients with and without type-2 diabetes. The results indicated that the proposed model is effective in clinical settings for type-2 diabetes, either early prediction or early prevention [8].

A. Dairi et al. [9] proposed an anomaly detection method for recognizing mental tasks using EEG signals. The authors exploited a deep learning model to detect normal and abnormal brain patterns during mental activities. The evaluation of the proposed model is performed on a publicly available EEG dataset, which achieved an overall accuracy of 98.31% above traditional classification methods. The proposed model is robust to any variation in noise levels and signal strength. This study suggests that the proposed method could be a practical tool for real-time recognition of mental tasks, potentially enabling applications, such as brain–computer interfaces for controlling external devices with mental commands [9]. Further, the medical data can be used for risk mortality prediction. Moreover, K. Nakamura et al. [10] proposed a deep learning model for predicting the risk of mortality due to heart failure. The authors utilized a time-varying covariate approach to account for changes in patient health over time, which leads to enhanced accuracy. The evaluation is performed on a large real-world heart failure dataset. The outperforming C-index is 0.741. Here, the model undergoes various conditions to demonstrate its robustness for various input features. It shows that the inclusion of time-varying covariates significantly improved the accuracy of the predictions. The proposed model could be valuable for clinical decision-making concerning heart failure patient management. It enables clinicians to identify high-risk patients and develop targeted treatment plans. The study suggests that deep learning algorithms can potentially improve risk stratification and decision-making in clinical practice for heart failure patients [10].

On the other hand, some researchers detect atrial fibrillation episodes based on 3D algebraic relationships between cardiac intervals. N. W. Qammar et al. [11] proposed a strategy based on the 3D phase space of the cardiac intervals and their algebraic relationships, which are modeled by the Grassmann manifold. The evaluation is carried out on 250 electrocardiogram (ECG) recordings with varying lengths and compared with two state-of-the-art methods for AF detection. The results show that the proposed method outperformed the two state-of-the-art methods in sensitivity, specificity, accuracy, and F1 score. The model is robust against noise and baseline drift, achieving a sensitivity of 97.56%, specificity of 99.15%, accuracy of 98.97%, and F1-score of 0.9804, which are higher than the two state-of-the-art methods. Their approach can be utilized for the early detection of AF episodes, leading to timely interventions and preventing complications associated with AF [11].

The use of medical data to enhance the accuracy of breast cancer diagnosis is widely recognized, as breast cancer remains the most prevalent cancer among women globally. For example, M. Yusoff et al. [12] systematically analyzed 25 papers published from 2018 to 2020 and evaluated their methods and performance metrics. The results showed that CNNs were the most used deep learning approach for breast cancer classification, followed by ensemble methods and recurrent neural networks (RNNs). The review also identified various data types used for classification, such as mammography images, histopathology images, and gene expression profiles. The evaluation uses different metrics, such as accuracy, sensitivity, specificity, F1-score, and area under the curve (AUC). The average accuracy of the reviewed methods ranged from 70% to 99%, with most achieving accuracy above 90%. The study also highlighted limitations and challenges in the reviewed papers, such as limited sample sizes, lack of standardized datasets, and difficulty interpreting deep learning models’ decision-making process. In conclusion, the article provides a comprehensive overview of the current state of deep learning methods in breast cancer classification and identifies potential areas for future research [12].
